# Using photovoice methods to explore older people's perceptions of respect and social inclusion in cities: Opportunities, challenges and solutions

**DOI:** 10.1016/j.ssmph.2016.09.004

**Published:** 2016-09-13

**Authors:** Sara Ronzi, Daniel Pope, Lois Orton, Nigel Bruce

**Affiliations:** Department of Public Health and Policy, University of Liverpool, Liverpool L69 3GB, United Kingdom

**Keywords:** UK, Older people, Aging, Age-friendly city, Photovoice, Community-based participatory research

## Abstract

Urbanisation and population ageing have contributed to recognise cities as important settings for healthy ageing. This paper considers opportunities, challenges and solutions of using photovoice methods for exploring how individuals perceive their cities and the contribution this makes to their health. It focuses on one aspect of older people's experiences – respect and social inclusion, in the context of a community-based participatory research. Drawing on selected findings (participants' photographs, associated quotes and researchers' field notes), we provide an assessment of the suitability of photovoice methodology for the intended purpose. Four groups of older people (n=26; aged 60 years or more) from four contrasting geographical areas in Liverpool, UK, were recruited purposively. Participants photographed perceived positive and negative aspects of respect and social inclusion in the city, reflecting on the meanings of the photographs in individual (n=23) and group interviews (n=9). Thematic and content analysis was conducted using NVivo 10 software.

The work reported here provides insights into how participants engage with the photovoice process; factors preventing taking photos of interest; and how photographs complement interviews and focus groups. The findings demonstrate that photovoice both facilitated the dissemination of personalised relevant knowledge, and encouraged critical dialogue between participants, and city stakeholders. Reported difficulties included photography of negative and social concepts, and anxiety when taking photographs due to (i) expectations of what is a ‘proper’ photograph, and (ii) the need to obtain consent from subjects. With preparation, training, and discussion of participants’ ideas not expressed through photographs, photovoice was well-suited to this topic, providing insights complementing other research methods. Through analysing the application of photovoice for exploring perceptions of respect and social inclusion in cities, our paper has identified potential issues and provides important recommendations for researchers on how photovoice methodology can be strengthened in exploring conditions for better health in the urban environment.

## Highlights

• The photovoice process enabled participants to reflect upon their city.
• Narratives and photographs provided a rich understanding of participants' views.
• Photovoice encouraged collective dialogue on aspects that matter to older people.
• Participants found it challenging to photograph negative and social concepts.
• Photography training, and building a relationship with participants are vital.

## 1 Introduction

“Photography helps people to see” (Berenice Abbott).

### 1.1 Background

Urbanisation and population ageing have contributed to recognise cities as important settings for healthy ageing. Due to physical and social changes associated with ageing, a supportive environment is especially important for older people's health and independence (Beard and Bloom, 2015, Yen et al., 2009). As older people (defined by the World Health Organisation (WHO) as aged 60 and over) tend to spend more time in their homes and local neighbourhood, the features of their neighbourhood can have a great influence on their day to day living, health and wellbeing; impacting upon their social inclusion or exclusion in that setting (Phillipson & Kendig, 2014).

To respond to the opportunities and challenges of an ageing population and increasing urbanisation, the WHO (World Health Organization, 2007) launched the Age Friendly City Initiative, proposing a set of eight interconnected domains that influence the extent to which individuals age healthily. In this context, an age-friendly city (AFC) is a community (comprised of wide-ranging categories of individuals, policies and services), which forms a physical and social environment that supports older people to live independently and contributing to various aspects of community life (World Health Organization, 2007). With advancing age, older people are more likely to be isolated and disconnected from their community (Phillipson, 2007). They are less likely to experience the health benefits of those living in socially inclusive environments providing social support and connectivity (Marmot, 2010). In the context of AFCs, supporting respect and social inclusion means encouraging people of all ages to cultivate social relationships, participate in the community, have access to resources, and feel valued and part of their community (World Health Organization, 2015).

### 1.2 Photovoice methods to explore older people's respect and social inclusion in cities

Although older peoples' involvement in decision-making processes within their neighbourhoods is acknowledged as important, this rarely happens in practice (Buffel et al., 2013). A number of methods using community-based participatory research (CBPR) – a research approach which comprises participation, action and collaborative inquiry often employed to solve urban health problems (Minkler, 2005) – are available for addressing the crucial question of ‘How do individuals and communities most effectively make their voices heard?’ (Buffel et al. 2013 p.54).

Visual methods, commonly employed in CBPR (Prosser & Loxley, 2008) as participatory visual research methods, have been increasingly developed in the field of health, social sciences, and education (Catalani and Minkler, 2010, Fraser and al Sayah, 2011, Pauwels, 2010, Rose, 2012a, Strack et al., 2004). These methods used to facilitate older people's participation, and to explore characteristics and issues of neighbourhoods/cities have included (i) photovoice (Alcock, 2011, Baker and Wang, 2006, Novek and Menec, 2013, Nykiforuk et al., 2011), (ii) participatory videos (Capstick & Ludwin, 2015), (iii) drawings (Scott, 2015), and (iv) photo-elicitation techniques (Pilcher, Martin & Williams, 2015). While photo-elicitation is an individualised visual research method wherein participant or research produced photographs are inserted into a research interview to explore a particular topic (Harper, 2002), photovoice is a predominantly collective visual research method wherein photographs taken by participants are used to explore and address community needs, stimulate individual empowerment, and create a critical dialogue to advocate community change (Hergenrather, 2009, Sanon et al., 2014). Among these methods, photovoice offers therefore a practical and accessible means of encouraging older people to identify community concerns and priorities, which can be brought to the attention of city stakeholders to stimulate policy change (Wang & Burris, 1997).

Publications incorporating photovoice methods have typically been descriptive, with most lacking an assessment on the comparative advantages and limitations of the process, and ways of dealing with these (Castleden et al., 2008, Drew and Guillemin, 2014, Evans-Agnew and Rosemberg, 2016, Harley, 2012, Pilcher et al., 2015, Prins, 2010, Switzer et al., 2015). Among the few studies that have considered these issues, Harley, 2012, Packard, 2008 and Prins (2010) critically discussed some ethical dilemmas experienced using the photovoice methods including: (i) balancing the power dynamics between researchers and participants; (ii) barriers to participants’ involvement, particularly with vulnerable people (e.g. homeless) and (iii) challenges related to asking consent for subjects who appear in the photographs. Prins (2010) discussed the ‘power’ of photography and its unintended consequences, highlighting that, depending on the context, the ‘innocent’ act of photographing can generate suspicion amongst community members. Hodgetts, Chamberlain, and Radley (2007) and Radley, Hodgetts, and Cullen (2005) examined the value of exploring photographs not taken by participants, calling for a more critical description of the entire photo-production process, which includes how participants engage with taking – or not taking – photographs. In the limitations of photovoice related to personal judgement, Wang et al. (1998) have also raised the issue of considering what the participant chooses not to photograph.

This limited discourse on photovoice methods employed with older people in exploring city issues, including how participants and researchers experience the photovoice process, and implications for interpreting the results, has important implications for its effective application in producing valid and comprehensive data, for example through the triangulation of transcripts from interviews, focus groups and photographs (Mays and Pope, 2000, Noble and Smith, 2015). As a consequence, there may also be limited efforts to refine the approach and techniques, for example in engaging with participants and city stakeholders to identify potential solutions (Catalani & Minkler, 2010).

### 1.3 Context and purpose of the study

The photovoice study reported in this paper was part of a wider study (doctoral) looking at approaches to evaluation of respect and social inclusion in older people in the context of an aspiring AFC. It included a qualitative study exploring factors shaping the ability of city stakeholders (defined as policy makers and service providers) to promote respect and social inclusion in an aspiring AFC (Liverpool, UK), which will be presented elsewhere.

Within this overall study, the photovoice component served to explore older people's perceptions of enablers and barriers to respect and social inclusion in the city, and to encourage them to find solutions to some of the issues identified. One key aspect of the mechanisms necessary to build AFCs is the need for local policy makers to involve and consider older people's views of the city in decision making and planning processes. Therefore the interaction between older people and city stakeholders was critical, which was facilitated by the photovoice process and the photo-exhibition event. This was the principal means of bringing older people's voices to the attention of city stakeholders, in a way that could encourage critical dialogue and stimulate policy and social change. Given the context of this study, it was not our intention to pursue extended cycles of photographs and discussions to further empower the group of older participants.

This study aimed to stimulate collective action and advocacy to affect policy and empowerment by (i) encouraging dialogue between older people and city stakeholders at the exhibition; and (ii) by ensuring that older people's views were brought to the attention of city stakeholders so that they could include their concerns in decision making and planning processes for an AFC.

Given the above, the objectives of this paper are to assess the strengths and limitations of photovoice methods to (i) effectively explore features of a city that promote or inhibit older people's perceptions of respect and social inclusion, and (ii) stimulate constructive dialogue between participants and city stakeholders. We provide an assessment of the photo-voice methodology based on our study, rather than a presentation of the findings from photovoice sessions, for which specific findings will be presented elsewhere.

## 2 Materials and methods

### 2.1 Photovoice methods

Photovoice, originally developed by Wang and Burris (1997), is a method used in CBPR, which focuses on individual and community assets, co-creation of knowledge, community building, individual and community empowerment, and combines research with action. Participants participate in group discussion and interviews, and use photography to document their experiences. The stories they tell about the photographs identify and represent issues of importance to them. Findings are usually disseminated through a photo-exhibition to promote community discussions, policy and social change (Wang et al., 1998). A description of each phase is given in Section 2.4.

### 2.2 Theoretical foundation

The theoretical foundation of photovoice is grounded in Paulo Freire's approach to education for critical consciousness (Freire, 1974) suggesting that the visual image was an important tool for enabling people to reflect about their community and also the contradictions within it. In doing so, individuals could become progressively aware of their own views of that reality, and cope with it. Three levels of critical consciousness that influence the way in which a person interprets and responds to the reality were identified; what Freire, 1970, Freire, 1974 called the process of ‘decoding’: the act of knowing. In the first level (magical level) the person is not conscious of the contradictions prevailing within the society, being perceived as something outside her/him, in which the “behaviours of passive adaptation actively contribute to his/her own oppression” (Freire, 1974 p. 837). In the second level there is a gradual perception of the societal reality along with its incongruities, but personalising of the problems and questioning of key issues, such as social injustice, is not present. Lastly, the greatest level of critical consciousness is represented by the individual's perception that his/her presumptions influence his/her perception of the reality. Here, the individual becomes “aware of” her/his “own responsibility for choices that either maintain or change that reality” (Freire, 1974 p. 837). Freire (1974) sought to stimulate individuals to discover and create their own learning through the practice. He used drawing to encourage collective reflection about community issues, leading participants to take action. According to Freire (Carlson et al., 2006 cited in Freire, 1970 p. 302), “dialogue can enhance reflection, understanding and action through a process of walking toward together while questioning”. Photovoice aims to engage research subjects in ‘seeing the world and transforming it’ (Wang & Burris, 1997). By taking photographs, the participant gradually becomes an ‘interpreter of the world’. In the current study, photovoice uses the participants' photographs and their associated stories to generate discussions about respect and social inclusion, and collective action and advocacy to impact on policy.

### 2.3 Research setting and participants

The city of Liverpool, in the North West of England, has recently initiated the process of becoming an AFC, and was chosen as the setting for this study. Four groups of older people (total sample: N=26) were recruited from four electoral wards; two representing the most deprived areas and two the least deprived areas in Liverpool (Jones & Mason, 2015); reflecting potentially different experiences of respect and social inclusion in the city, for which specific findings will be presented elsewhere. Older people in the most deprived areas are known to experience worse health, social exclusion, and poorer access to services and support, compared to those residing in more affluent areas (Marmot, 2010). Life expectancy is also worse in more deprived areas (Liverpool having an almost 10 year difference in life expectancy between the most and least deprived areas) (Jones & Mason, 2015). Moreover, the likelihood of experiencing social exclusion increases with age (Scharf & Keating, 2012).

The four geographical areas were accessed through connections developed by the author with ‘gatekeepers’ working in local community organisations. Gatekeepers were provided with details of study inclusion criteria (Table 1) and assisted with the invitation of participants.

**Table 1:** Inclusion criteria.

1. **Being able to consent for themselves.**
2. **Being an older person aged 60 or more.**
3. **Being able to speak English.**
4. **Living in Liverpool.**
5. **Being British or having lived in the UK for at least 10 years.**
6. **Being able to manage digital cameras and take pictures about the topic under study.**
7. **Being able to attend and participate in group meetings and interviews.**

### 2.4 Procedures

Data were collected between September 2014 and March 2015, with the process of building relationships with older people starting in June 2014.

Eight months prior to the start of data collection, existing organisations interested in older people in Liverpool were approached by [SR] during targeted local events on ageing. A relationship of trust with city stakeholders was built over one year (February 2014-May 2015), wherein [SR] informally explained the project, kept them informed about the progress with the study, and subsequently invited them to a photo-exhibition of the material collected through this study. This strategy/process resulted in the majority of invited stakeholders attending the public exhibition.

Each photovoice project lasted approximately one month. Each group of participants participated in two focus groups (approximately one-two hours) and an individual semi-structured interview (approximately fifteen minutes to an hour). These were audio recorded with permission. At the end of the study, participants received a supermarket voucher (£20) to compensate them for their time. Given the context of our study, and based on [SR's] experience in conducting previous photovoice projects with older people, this duration was deemed as appropriate to collect data and keep the participants involved, as well as being able to create a platform to encourage critical dialogue between older people and city stakeholders.

**Fig. 1:**
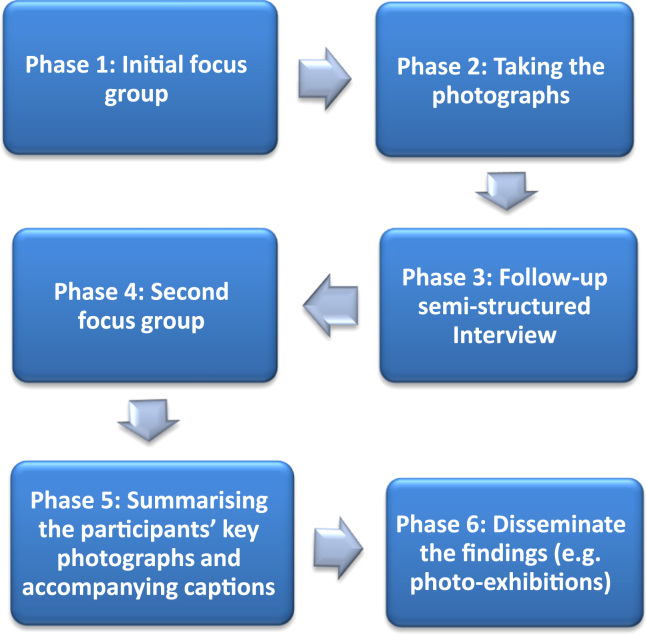
Phases of the photovoice process adopted in this study. Adapted from Nykiforuk et al. (2011).

The photovoice project had 6 phases (Fig. 1):

1. An initial focus group (n=5 focus groups; one group had 2 initial focus groups with different participants) introduced the project, photography and ethical training. Participants then received digital cameras, were given a broad pre-identified problem to be explored (respect and social inclusion). They were asked to photograph aspects of Liverpool that they felt ‘enabled or prevented them to feel valued and part of the community’ and to identify potential solutions to any problems identified. To limit the researcher influence over subject matter for participants' photographs, the photography task was kept very general. Participants were left free to take any object/person/place that referred to respect and social inclusion in the City.
2. Participants took photographs over a period of a week.
3. Semi-structured interviews were conducted (n=21 individual interviews; n=2 interviews were conducted in pairs, with a total N=25 participants being interviewed). During interview, photographs were displayed on a laptop and shown to each participant who was requested to select the most meaningful (between three and six) to discuss in a subsequent focus group. Participants, who had not seen the photographs they had taken before the interview, selected 127 photographs in total. Each participant discussed the meanings of the photographs using the SHOWeD technique (Wang et al., 1998, Wang and Burris, 1997). The SHOWeD technique consists of different questions that relate to the photograph: What do you See here? What's really Happening here? How does this relate to Our lives? Why does this problem, concern, or strength Exist? What can we Do about it? Additional questions were adapted from previous literature on ageing, and from two prior photovoice studies exploring perceptions of ageing conducted by Ronzi (2013) (Appendix A). After exploring the photographs taken and the participant's stories associated to these, we asked if there was any photo that the participant wanted to take but that for various reasons they did not take, so as to generate discussion on aspects that were not photographed. Further, we asked participants how they experienced the photovoice process and the overall project (e.g. How did you find the project? What did you like most and least about the project? What can I improve next time?).
4. Participants discussed their photographs during a second focus group (n=4). Participants were asked how they wanted that the findings were communicated to relevant people in the City. We suggested a photo-exhibition, and participants highly welcomed the idea. At the end of the focus group we asked participants how they experienced the photovoice project and what could have been improved in future projects.
5. A brief summary was written based on the participant's original description of the photograph (from interview and focus group transcripts) to accompany each photograph. To ensure that these stories reflected the intentions of the participant, [SR] showed all the photographs and associated summaries to each participant, who reviewed and edited the final summary and title prior to being printed and displayed in the photo-exhibition (Foster-Fishman et al., 2010). For this occasion, [SR] asked participants to review and agree on (i) the choice of the photographs and accompanying captions to display in the photo-exhibition; and (ii) the different sub-headings used to group photographs and accompanying captions in the photo-exhibition (e.g. access to public transportation, access to green spaces, etc.). Due to limited space at the Museum, 60 out of the 127 photographs were displayed. This guaranteed that all the photographs that the participants thought were most meaningful were included (participants had between 2 and 3 photos displayed). The researcher team was responsible for the printing of the photographs and for their display at the Museum.
6. An exhibition of the older people's photographs took place in May 2015 in a landmark community setting – the Museum of Liverpool. The aim of the exhibition was to provide a forum to disseminate study findings, and to encourage critical dialogue amongst invited participants, city stakeholders, researchers and members of the community about those aspects of the city that were perceived to be important to older people, and to influence policy and social change. In total 71 people attended the event. Speakers included a representative from the council (mayoral lead for older people), an executive of the museum, and two leading academics from the university. We assessed the event with a short evaluation survey and observation notes were taken by five researchers.

### 2.5 Ethics

Ethical approval was granted by University of Liverpool in July 2014. All participants were provided with written information and signed a written consent form at the start of the initial meeting. Multiple consent forms were used, including (i) the participant's consent to take part in the study, (ii) acknowledgment and release forms – for people who appeared in any photograph, and (iii) a form for release of the photographs asking permission to use participants’ photographs in dissemination of results (Wang & Redwood-Jones, 2001). We have followed the ethics guidance developed by Wang & Redwood-Jones (2001), Strack et al. (2004) and Novek, Morris-Oswald, and Menec (2011).

Photovoice requires consideration of various ethical implications related to the photographs, including photo ownership and individuals appearing in the photographs, which were explained during the initial meeting. Participants were given consent forms and asked to obtain written consent from people who appeared in the photographs. Participants were instructed to inform every person who appeared in the photographs on the purposes of the study and that the photographers were to be used as a part of a doctoral thesis, to write publications and make photo-exhibitions. We clarified to participants the ‘rules’ on when consent was needed (e.g. individual or group is ‘featured’) and when not (where people can be regarded as a crowd), so to allow participants to take photos of topics involving people more easily (Wang & Redwood-Jones, 2001).We were aware of the ethical implications related to individuals under 18, with respect to the pictures taken by participants. Participants were also advised not to take photographs of people under 18, unless they are family members or close friends. At the interview, we asked participants to sign a form for release of the photographs asking permission to use their photographs in dissemination of results. This was an important aspect to consider, as being the owners of the photographs, participants have the right to have their voices heard through the representation of photographs and accompanying captions as they feel appropriate (Evans-Agnew & Rosemberg, 2016).

### 2.6 Data analysis

This study sought to explore perceptions and meanings that older people have of respect and social inclusion in the urban context, within a CBPR methodology. Therefore, this study was rooted in the interpretive tradition aimed at exploring individuals’ interpretation of reality. Within the interpretive approaches, interpretive phenomenology was chosen as the most appropriate approach to guide our analysis. With this approach, data are interrogated with respect to the meaning people attach to their experiences of social reality (Green & Thorogood, 2009).

**Fig. 2:**
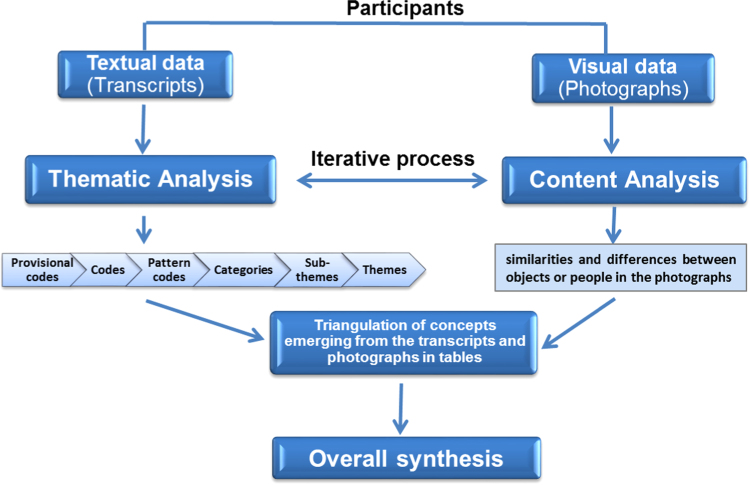
Flow chart of the overall data analysis process.

Analysis was conducted of text and photographs in parallel, with iterative cross-referencing of emerging themes. Fig. 2 shows the relationship between the different elements of the data analysis process.

Content analysis of the photographs explored similarities and differences between objects or people portrayed by the participants and aimed to complement the thematic analysis (Rose, 2012). Interviews (n=21 individual interviews; n=2 interviews were conducted in pairs. N=25 participants were interviewed) and group discussions (n=9) were transcribed verbatim and coded using NVivo 10 qualitative analysis software (QSR International, 2014). Thematic analysis of the transcripts identified themes and sub-themes from data (Miles & Huberman, 1994). Initially, transcripts were reviewed one-by-one, and provisional codes were applied to the transcripts based on the topic of respect and social inclusion in cities; codes were iteratively refined and categorised. Subsequently, transcripts were analysed more deeply, and categories were collated into themes and sub-themes emerging in the text. Themes and sub-themes' names were refined and agreed by the research team. SR read, coded, and analysed the transcripts, for which 20% of the transcripts were double coded by a member of the team. The codes were compared, and any discrepancy that arose was discussed. Four researchers were involved in the process of analysis, which was iterative, with sub-themes and themes that were constantly refined as the analysis progressed. We then triangulated concepts emerging from the transcripts and photographs in tables: analysing each photograph in relation to the meaning(s) that the participant attached to it (Keats, 2009). Each photograph was then allocated themes and sub-themes identified through the thematic analysis, with supporting quotes referring to the photograph. Table 2 shows an example of this integration of concepts emerging from a photograph with related quotes used to define and reinforce the main findings.

**Table 2:** Example of integration of concepts emerging from the photograph and quotes (from P15) arising from the interview and focus group discussion (Fig. 1); integration of these sources of data was used to define and reinforce the main findings.

Supporting information [Quotes]	Photograph [Title]**: Access to museums**
Voice of the photographer represented by selected text from participant's description of photograph which was checked and agreed with participant	**Central Library, Liverpool**

(P15 – Phase 3 interview)	
**“This is the library. I like books and inside it's absolutely beautiful… we have such as lovely facility here […]it's lovely to have a look and see whatever you want to see… it's open for anyone in Liverpool to go in, so it's not local community but it's for the community of Liverpool”. (P15, IV) (Central Library, Liverpool)**	
Other quotes related to the same photograph (P15 Phase 4 focus group):	
**“This is the Central Library, and a) the building is lovely, and b) anyone can go in. […] It's lovely to have a look and see whatever you want to see”. (P15, FG2) (Central Library, Liverpool)**	
**“I just like going around […] we have such as lovely facility here. […]”. (P15, FG2) (Central Library, Liverpool)**	

Overall synthesis [themes and sub-themes]	

Theme: **Enablers to respect and social inclusion**	

Sub-themes **(obtained from thematic analysis of all transcripts) to which content of this image relates:**	

• **Access to libraries**	

• **Access to learning opportunities**	

• **Symbol of Liverpool**	

• **Asset in the community**	

## 3 Results

This section presents selected findings on the opportunities, challenges and solutions of using photovoice methods to (i) effectively explore features of Liverpool City that promote or inhibit older people's respect and social inclusion, and (ii) convey to city stakeholders those aspects that were perceived to be important to older people. It presents a description of the following sections: the photo-production process as a way to raise participants' consciousness; factors influencing participants’ ability to take photographs; and overcoming some of the challenges. A total of 26 older people participated in the study. Demographic details are shown in Table 3.

**Table 3:** Details of participants taking part in the photovoice study.

Group	Geographical area	Level of deprivation	N	Gender	Age group	Ethnic background
				(M=male; F=female)		
**1**	A	High	10 (n.1 participant attended the 1st focus group only; n.1 participant did not attend the second focus group)	3M, 7F		5 White British,
					60–70: 4	2 Asian British: (Chinese, Pakistani)
					70–80: 3	
						3 Black British
						(2 Black African, 1 Caribbean)
					>80: 3	
						
**2**	B	Low	4	2M, 2F	60-64: 1	4 White British
					65-70: 2	
					70-75: 1	
						
**3**	C	Low	6 (n.1 participant did not attend the second focus group)	2M, 4F	60–64: 2	5 White British,
					75–80: 2	1 Other White Background: Italian
					80–85: 1	
					>85: 1	
						
**4**	D	High	6 (n. 1 participant attended the 1st focus group only)	6F	65–70: 1	6 White British
					70–75: 3	
					75–80: 1	
					>80: 1	
Total			26	7M, 19F		

Four participants partly completed the project due to illness and/or medical appointments. To allow for dropouts, a greater number of participants were recruited than originally required, to ensure that each group had between 4 and 8 participants completing the project. Among those who partly completed the project, 2 participated in an additional interview including questions asked during the second focus group.

The ethical principles of CBPR emphasise commitment to include participants whose voices are often ignored (Centre for Social Justice and Community Action Durham University – National Co-ordinating Centre for Public Engagement 2012). Four people with limited mobility (e.g. use of the walker and walking sticks), participated in the study. Older people were told that they could be accompanied to take photographs if they needed assistance. SR accompanied three participants to places where they wanted to take photographs, and prepared the cameras for them to take photographs.

In the analysis, we compared transcripts from interviews and focus groups to see if there were differences between groups. We found consistent views among participants about how they experienced the photovoice process. However, differences were noted about the time spent to build trusting relationships with participants. From the reflexivity notes taken by [SR], building a trusting a relationship was more challenging and required longer time with participants living in more deprived geographical areas, than those living in more affluent geographical areas.

### 3.1 The photo-production process as a way to raise participants' consciousness

The majority of participants stated that photovoice invited them to effectively engage with the photographs, with the collective discussions offering an opportunity to critically think as a group about various aspects of their city.

“I have enjoyed the interactions with other people, your focus groups, and it was interesting for me to find things to photograph. I really thought: what is important for me as an older person?” (P3 IV).

The photo-production process facilitated participants to become more aware of some features of the city which contributed to or prevented their respect and social inclusion, as illustrated by the following quotes and researchers' observations. These quotes illustrate how the photovoice process increased awareness at the individual level, and made participants break with their habitual ways of thinking, and to see beyond what they started to take for granted.

“It's the city we have lived in so long! It did make you thinking…” (P13 FG2).

“Taking pictures was quite good […] I had to look at things and I thought ‘what can I do there?’ and it makes you thinking a bit.” (P11 FG2).

“It actually made me thinking on what I want from Liverpool as an older person”. (P3 IV).

**Fig. 3:**
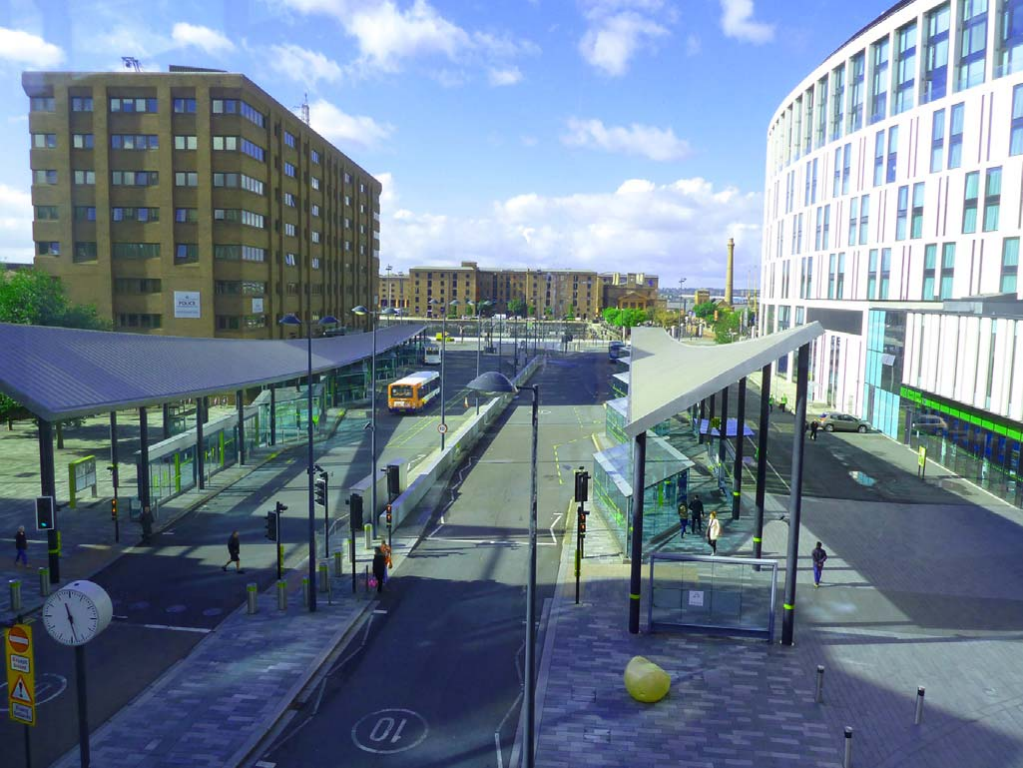
Example of a perceived barrier to respect and social inclusion.

Through the photographs, participants identified some barriers to respect and social inclusion in the city, and ways to address some of the issues identified. An example is shown in Fig. 3, where the participant portrayed a station considered very uncomfortable due to lack of protection from the wind.

“Growing older is […] about keeping the opportunities the same for you as they are for everybody else. So things like this [transportation] become very important. As you can see, Liverpool One bus station is completely open, and wind can still get in! We should use the learning from South Parkway station, and apply it to this station, so that you’re behind closed doors, in comfort, while waiting for a bus.” (P1) (Liverpool One bus station)

In Fig. 3 the participant was concerned about how some aspects of the City were not being considered by local policy makers. The participant then went on to illustrate how this could be remedied.

**Fig. 4:**
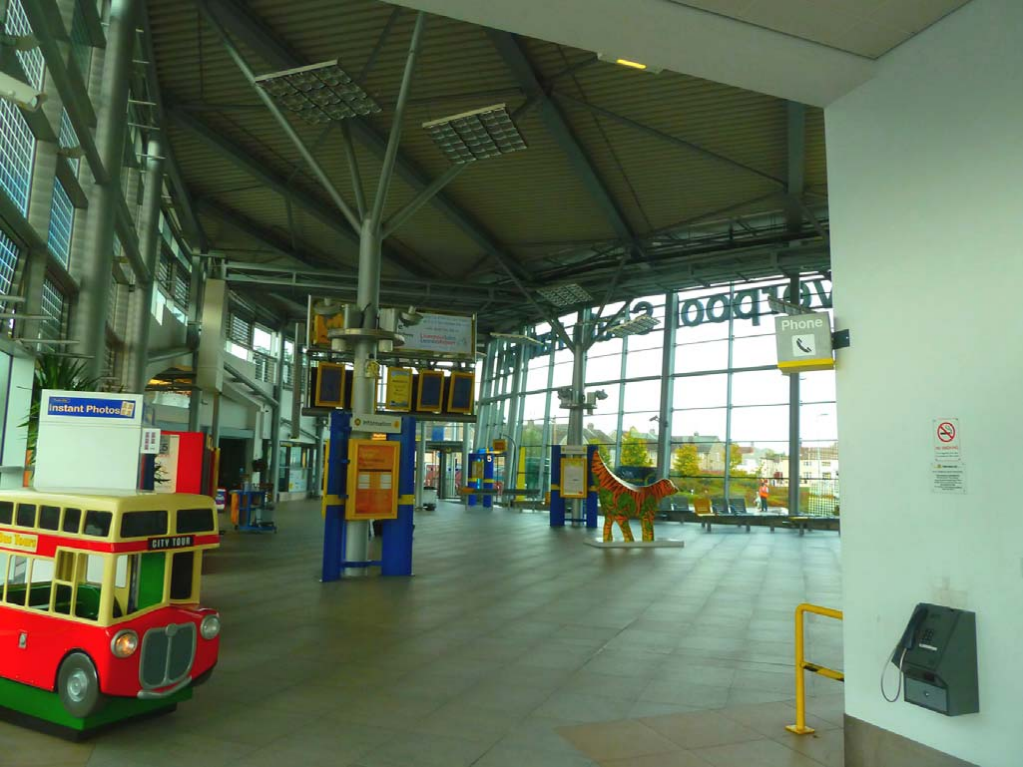
Example of a perceived enabler and solution to respect and social inclusion.

In the second photograph (Fig. 4) the participant suggested how these negative aspects could be improved. He identified an enclosed bus and train station as an enabler to respect and social inclusion.

“I am very lucky because I have got all of that near to me, and it gives me access to a lot of options to travel. It just makes it so much easier for people to travel. Once you get inside the building, you’re protected from the weather and the wind, it is very cosy, convenient, and very accessible.” (P1) (South Parkway Railway Station)

It would have been difficult to convey the same rich information on what aspects the participant considers important using other qualitative methods such as interviews or focus groups. The photographs provide a deeper level of detail on the context that helps ‘to see’ and explore the participant's perceptions of the city. Indeed, in Fig. 3, Fig. 4 the participant described the scene to help highlight what aspects were important to him in the photographs (e.g. in the first station the glass and canopies are unable to protect users by the wind, while the second station is behind closed doors).

The photographs and accompanying captions also allowed direct communication of what was meaningful to the participants through the photo-exhibition, and attendees highlighted that some participants engaged with visitors in explaining their own photographs.

“Seeing photo-voice participants explain their pictures to other visitors” (Anonymous attendee).

The fact that some participants spontaneously explained their photographs to city stakeholders suggested that the photovoice approach had truly engaged participants with the study, and empowered participants’ voice. Related to this, some participants reported a sense of ‘ownership’ over some of the photographs, saying, for example, ‘that was mine’ (no photographs had names). In doing so, the photo-voice process stimulated participants to express their perceptions to the attendees.

Some (anonymous) comments provided by attendees on the photo-exhibition reported that the photographs and accompanying captions provided a greater impact than could be achieved with traditional reports. These comments demonstrate an increased awareness at the community level as a result of the photovoice process.

“The photographs clearly show the life in Liverpool and how older adults engage with it. Fantastic!”(Academic).

“An excellent cross-section of how different people see our city.” (Participant).

Further (anonymous) comments provided by attendees noted that the photo-exhibition event managed to create a forum where participants, academics and city stakeholders met together to discuss older people's views of the City.

“Very nice to see an example of research that truly connects the University with its surrounding community.” (Academic).

“Getting people from the community, museum, and university together.” (Clinical commissioning group representative).

### 3.2 Factors influencing the participants' ability to take the photographs

**Fig. 5:**
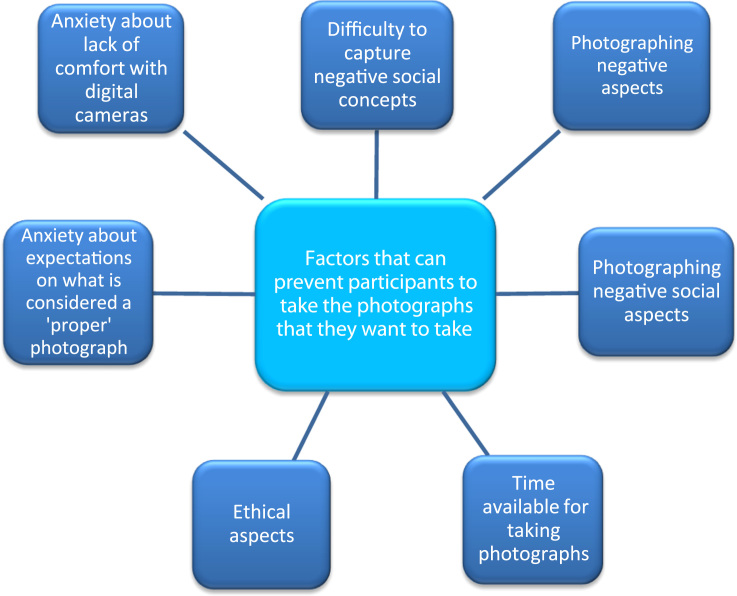
Factors that can prevent participants taking photographs that they want to take, as identified in this study.

Connected with the photo-production process, most participants were able to take photographs of enablers and barriers in the city. However, a small number of participants reported some challenges in finding suitable images to represent the constructs they wished to portray identified during discussions in the second focus group summarised in Fig. 5.

### 3.3 Photographing negative aspects

Overall, participants took more photographs of positive, than negative aspects of the city. The reasons for taking fewer negative photographs included: participants not perceiving many negative aspects, and wanting to portray their city in a positive light. In this quote, a participant considered that individuals prefer thinking of positive aspects:

“We don't like to think things being negative of course, […] but there are negative things.” (P13 FG2).

This might not represent an issue of itself, but if participants were unable to identify negative aspects despite being aware of them, potentially important perspectives of their city would be missed. A connected explanation is that many participants used photography to promote awareness of various facilities that were available contributing to respect and social inclusion in Liverpool.

“It costs nothing to go to and it's a very nice, tranquil and beautiful park. I think that should be promoted because people would go and enjoy the gardens.” (P11) (International Festival Gardens, Liverpool).

**Fig. 6:**
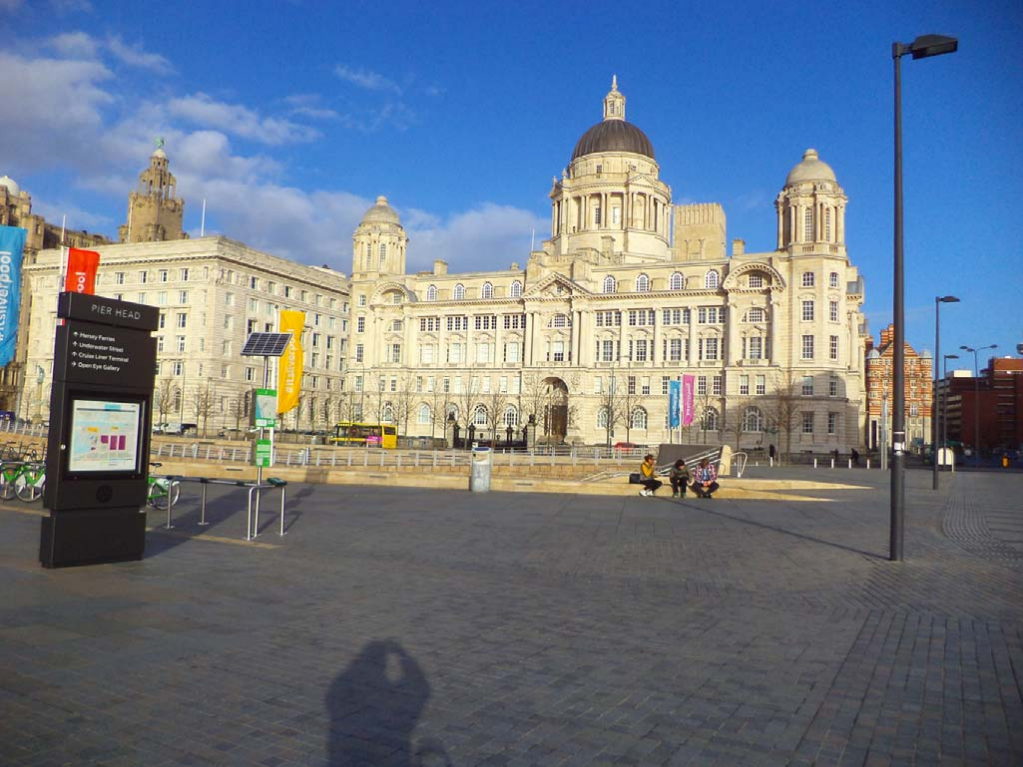
Example of a perceived enabler to respect and social inclusion.

In this example the participant used photography to ‘signpost’ a facility in the city, so that more people could be aware of it. Fig. 6 further indicates that since some participants wanted to promote awareness of those places for which they recognised a strong sense of pride and identity, this might explain why more positive aspects emerged.

“Wouldn’t that engender pride in anyone? I think that’s just beautiful. The feel-good factor […] I think everyone should see how magnificent this place is. Just living in an area that has such buildings is wonderful.” (P20) (Pier Head, Liverpool)

### 3.4 Photographing negative social concepts

**Fig. 7:**
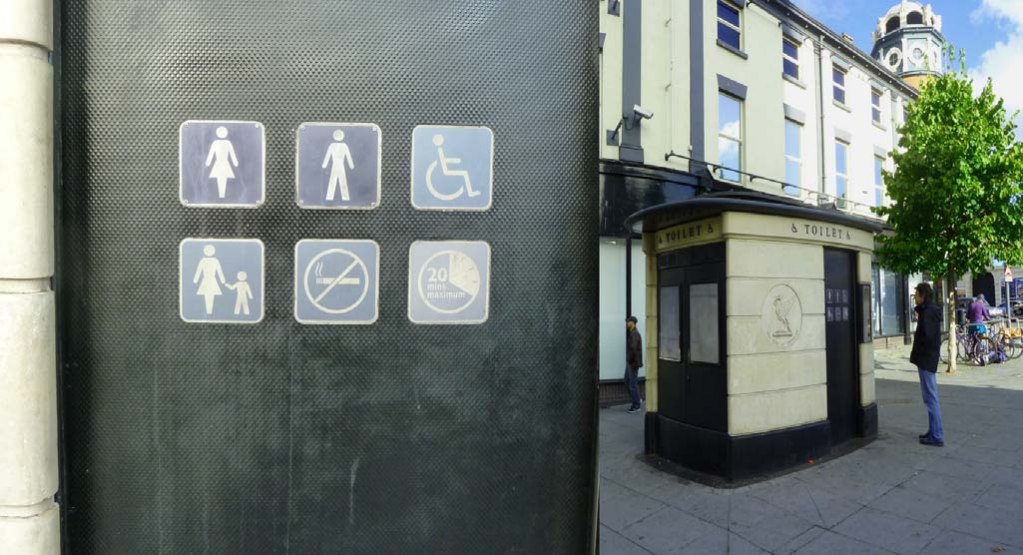
Example of a perceived negative social aspect.

Some participants reported that they found it most difficult to take photographs of negative social concepts (e.g. social isolation) rather than negative physical aspects (e.g. rubbish in the street) or positive social concepts (e.g. social participation). However, a few participants managed to capture negative social concepts; Fig. 7 provides an example of this.

“This toilet’s not inviting, it’s not accessible…and I’ll be a bit anxious about getting locked in. It’s counterproductive to meeting the needs of older people. Your body is changing and you’ve different needs…, but you need immediate access to clean toilets. It’s against social inclusion. It’s a barrier because no many older people will be confident to go to town if there’re not enough accessible public toilets.” (P1, IV) (Bold Street, Liverpool)

The following quote shows an important ‘social’ concept identified by one participant at interview. However, the participant was not able to represent this concept in a photograph.

“Not just in Liverpool, within Western culture, we don’t value older people. It's almost like that you retire, and you don’t work anymore for money, so you have no value in society; while we have a great deal of resources up here. […] we have a great deal of knowledge and wisdom that we could impart to young people if they want to listen at us”. (P3, IV).

### 3.5 Time period for taking photographs

As noted in Section 2.4, we discussed with participants photographs taken and untaken, and this was useful to generate group discussion about some aspects not portrayed in the photographs. During the second focus group, participants were asked what they liked least and most about the project. Some participants reported that time constraints were another reason for recording fewer negative and social aspects:

“Some of the vandalism, which does happen, and just didn't happen when I had the camera!” (P15 FG2).

This shows that some participants might have been unable to photograph some negative aspects due to these not happening during the timeframe for taking photographs. Participants reported that they wanted to capture recent episodes of vandalism, not just evidence of past acts of vandalism. This episode stimulated other participants to comment on recent episodes of vandalism in the City, even if they did not take any photograph of it. This represents an important example in which exploring aspects of photographs not taken (in this case vandalism) allowed us to identify important concerns that some older participants had about the city. Similarly, the quote below shows that extra time might have allowed more aspects of the city to be portrayed, not just negative aspects.

“I could have taken hundreds of pictures! […] some of the museums and the art galleries […] I would have liked to have told you more about Liverpool in photographs; I may do that anyway for myself.” (P21 IV).

One week to take photographs was considered an appropriate time to keep participants engaged, and to complete the task. It was also important to ensure that participants remembered clearly the meanings of the photographs, since they were not provided with any journal/diary to act as a prompt.

### 3.6 Ethical aspects

During the photography training the importance of gaining informed consent prior to taking photographs of individuals was stressed. This was successful, as all participants who took photographs of people obtained the required signed consent. However, given that the study explored respect and social inclusion in the urban context, there were not many photographs portraying individuals. In this quote, a participant wished to have captured some aspects of Liverpool spontaneously, without the need to asking consent.

“I would have liked to take photographs of Liverpool just doing ordinary things without them knowing that I was taking pictures…like someone singing outside one of the big stores… kids dancing in the music.” (P21 IV).

The requirement for consent might therefore have prevented some participants taking photographs of individuals, and some participants' perspectives would have been missed.

### 3.7 Social expectations related to taking photographs and comfort with using the camera

Minimum guidance was given concerning what aspects to photograph, and participants were left free to photograph aspects that were most meaningful to them. However, when photographs were uploaded onto a laptop, some participants expressed feelings of anxiety to please, for example saying “I hope that I took the pictures that you wanted”. This relates to wanting to please the researcher by taking photographs of what the participant considers appropriate to show for the project, rather than what the participant perceives as worth photographing.

“I was thinking to take a picture of my grandson and daughter but then I thought; it's too obvious! Obviously they’re important to me… but it's too obvious so I didn't take it”. (P3 IV).

This example shows that the pressure to take the ‘proper’ photographs might have prevented some participants from photographing some aspects that were meaningful to them. Besides, some participants expressed feelings of expectations about their technical ability with the camera, as illustrated by these quotes:

“I should have taken one of inside [Central library]” (P16, IV).

“There could be better pictures of that [International Festival Gardens]” (P12, IV).

“The picture just doesn't do it [Japanese garden] justice, to be honest” (P15, IV).

One reason for this could be that some participants were not familiar with the use of digital cameras (some of them have never used one before, or the cameras used previously were not digital). Although participants were reassured throughout the research process, the lack of familiarity with digital cameras might have led participants to feel that taking ‘proper’ photographs was beyond their ability.

### 3.8 Overcoming some of the challenges

#### 3.8.1 The ‘missing photographs’

The participants' untaken photographs were explored at the interview, which made it possible to identify those aspects that were not photographed (the ‘missing photographs’), and to generate discussion on topics that were not otherwise addressed by the research. In the example below, one participant did not take photographs of barriers to social inclusion, but when prompted to reflect on why, new evidence on this construct emerged.

“Some of the negative aspects that I thought you can't take a picture of are: I don't know my neighbourhoods or […] of somebody who is isolated or lonely.” (P17 FG2).

Asking about untaken photographs encouraged one participant to subsequently take the photograph that she had wanted to take (in this case, a tree outside her road that she was caring for). After meeting with the participant to check the captions accompanying each of her photographs, she spontaneously showed a photograph recorded on her camera of ‘that tree’ mentioned in her interview. She reported that planting flowers surrounding the tree enabled her to speak to neighbours.

“You were asking how we could make the community better […]; I felt that by doing that, I was going out into the community to give pleasure to the community […]. I've lived there 41 years, and I've spoken to people out there that I'd never spoken to before since.” (P19 IV2).

These quotes indicate that exploring untaken photographs can reveal very important aspects that would otherwise not have been available to the research.

### 3.9 Photography training

Training in photography was important in addressing other potential challenges. Since there was uncertainty in how familiar participants were in taking photographs, simple digital cameras were selected with large, clear controls and default automatic operation. Older people were shown how to use the cameras during the recruitment process and first focus group. Participants were also provided with written instructions and a reminder of the photography mission. These steps were important, particularly to reassure those participants who showed some initial ‘scepticism’ about their ability to use cameras.

Participants were reassured that the researchers were interested in exploring the ‘stories’ within the photographs, so that they could focus on the content of the photograph, rather than on its beauty. At the end of the study, the initial scepticism felt by some participants turned into a sense of ‘pride’ regarding the photographs taken.

“Initially I thought: ‘oh no, that does not really apply to me’… but actually it did it in so many ways! I thought ‘I would just pass that’, but then I enjoyed!” (P10 FG2).

“I think people get frightened of new things, and you’re saying to the group ‘you have to take these photographs and you have to discuss in a group’, and you’re: ‘oh!’, and that's fear […] but once we started it was alright.” (P11 FG2).

These quotes show that the photography training and ongoing support are essential to make participants comfortable with the entire photo-production process.

## 4 Discussion

### 4.1 Overview of main findings

These findings demonstrate the utility of photovoice in facilitating (i) older people to articulate what was most important for their respect and social inclusion in their city, and (ii) the dissemination of findings to city stakeholders. Among the main challenges encountered were anxiety to please, difficulties in photographing negative social concepts, and the need to obtain permission of subjects. Discussing photographs that were not taken, and photography training, could overcome these challenges.

This study has addressed the paucity of assessment of some of the strengths and limitations of the photovoice process as described by various authors (Evans-Agnew and Rosemberg, 2016, Harley, 2012, Hodgetts et al., 2011, Prins, 2010, Switzer et al., 2015). This has included whether and how effectively photovoice helped to effectively explore city issues of respect and social inclusion and to stimulate constructive dialogue between participants and city stakeholders. In addition this study has identified potential issues and solutions that would be common to other research areas using photovoice methods. This study adds to the current knowledge on CBPR and visual methods used in public health to engage individuals in exploring the urban context, and to encourage individual and community empowerment and policy change.

### 4.2 Challenges and solutions of photovoice methods

In our study, some of the participants expressed anxiety in taking what they perceived to be ‘proper’ photographs. Evans-Agnew and Rosemberg (2016) have raised the issue of the influence that researchers can have over the participants’ choice of photographs. This aspect needs to be considered, as participants may want to please the researchers by taking photographs that reflect more what [they believe that] the researchers would like to see, rather than the participants' own experience. In a study conducted by Prins (2010) some participants reported being indecisive on what to photograph, and being embarrassed to be seen taking photographs by other community members. This either stopped them taking photographs, or involved participants asking others (e.g. relatives) to take photographs for them. However, this study took place in a rural community characterised by post-war fear and mistrust, and taking photographs might have been seen as a violation of local social norms. In the current study some participants raised concerns due to their social expectations for taking photographs and comfort using the camera. Therefore ensuring participants are comfortable with the photo-production process is important.

Some of our participants faced challenges in photographing negative social concepts. Previous studies have attempted to overcome this by encouraging participants to take photographs of both physical and social aspects of their cities (Novek et al., 2011). Novek et al. (2011) stressed that older people photographed more tangible aspects, but also social aspects of the city were portrayed (e.g. vandalism). In the current study participants captured some similar social concepts (e.g. intergenerational relationships and safety issues in the neighbourhoods). However, the main challenge for our participants was capturing negative social concepts rather than positive ones. Although Novek et al. (2011) described many positive social aspects of the city, and some negative physical aspects (e.g. sidewalks), they did not discuss the issue of barriers experienced by older people in portraying negative social issues. This may partly be due to their diverse photography mission, which aimed to capture age and non-age-friendly features in cities. The current study focused on features of respect and social inclusion in the city – which included an important focus on the social environment.

In the current study, most of the negative and/or social aspects not taken in photographs emerged during the interview, when we asked participants if there were photographs that they wanted to take but that for various reasons they did not. Based on our study, we argue that exploring untaken photographs reflects more an individual experience that can be discussed at the interview. However, exploring untaken photographs can also help to generate group discussion. In our study, this emerged when some participants reported that more time would have allowed them to take some photographs of specific aspects of the City (e.g. vandalism) that they wanted to take. This prompted other participants to discuss related topics during the focus group. Therefore, exploring untaken photographs can: (i) encourage the participant to reflect a more personal experience at the interview; and (ii) stimulate discussion in focus groups about aspects not identified in the photographs and/or in participants' narratives. In our study, discussing photographs that were not taken with participants ensured that we covered views on respect and social inclusion which were not captured in the photographs, and helped to identify potential limitations of the method as used. This approach was recommended by Hodgetts et al. (2007) – researchers using photovoice methods should also incorporate this aspect in their methodology. In this study, time constraints were reported as another reason for identifying fewer negative and social aspects. Since participants did not use diaries/journals that could act as a prompt, we considered one week sufficient time to complete the task. A review by Catalani and Minkler (2010) reported that the most participatory photovoice studies engaged with participants in several cycles of taking photographs and discussions. This can be a way of addressing time constraints and participants' difficulties in taking certain photographs. However, given the context of this study, it was not our intention to pursue an extended process of cycles of photographs and discussions, and this was based on a combination of pragmatic reasons. This study aimed to create a platform to encourage dialogue between older people and city stakeholders; and to bring older people's views to their attention of city stakeholders so that they could include their concerns in decision making processes. We also wanted to minimise the risk of drop-outs, given various chronic and mobility issues experienced by participants. Maintaining older people's involvement in the long term throughout the study can be challenging, and this aspect has been raised by Novek et al. (2011) as well. We are aware that a longer time for photo sharing may be desired. However, as this research incorporated a study of city stakeholder perspectives and the goal of initiating dialogue, we prioritised a photo sharing event where participants, city stakeholders, researchers, and the public, could come together and initiate a collective dialogue to achieve an impact on AFC policy.

In this study, the photography training addressed some of difficulties experienced in the photovoice process. These included: social expectations related to taking photographs and comfort using the camera, ethical implications related to asking written consent to individuals appearing in the photographs. The Catalani and Minkler (2010) review reported that the most participatory photovoice studies had intense photography training. For example, participants can be taught how to photograph challenging social issues. However, focusing on a particular way of taking photographs may alter the participants' ability to discover further ways to observe the world and learn photography through practice. In their discussion of the importance of covering technical and ethical aspects of photovoice, Wang and Burris (1997), stressed participants should continue to improve their abilities, confidence and understanding through the photovoice process, rather than through more intensive training. We agree with Wang and Burris (1997) that a photographic training about ethical aspects, and how to use cameras is essential, but it should limit the influence of the researchers over the choice of photographs taken by participants (e.g. it should keep a very general ‘photographic mission’). Many authors have discussed ethical considerations for CBPR and visual methods (Banks and Armstrong, 2012, Evans-Agnew and Rosemberg, 2016, Rose, 2012c, Wang and Redwood-Jones, 2001). Harley (2012) and Evans-Agnew and Rosemberg (2016) noted that many photovoice papers did not offer provide adequate description of the ethical process for participants and researchers. In our study, we carefully discussed ethics and consent with participants. Wang and Redwood-Jones (2001) have suggested that training should cover rules on images ethics. Therefore, clarifying, as part of the training, the ‘rules’ on when consent is needed (e.g. individual or group is ‘featured’) and when not (where people can be regarded as a crowd), might allow participants to take photos of topics involving people more easily.

### 4.3 Opportunities of photovoice methods

**Fig. 8:**
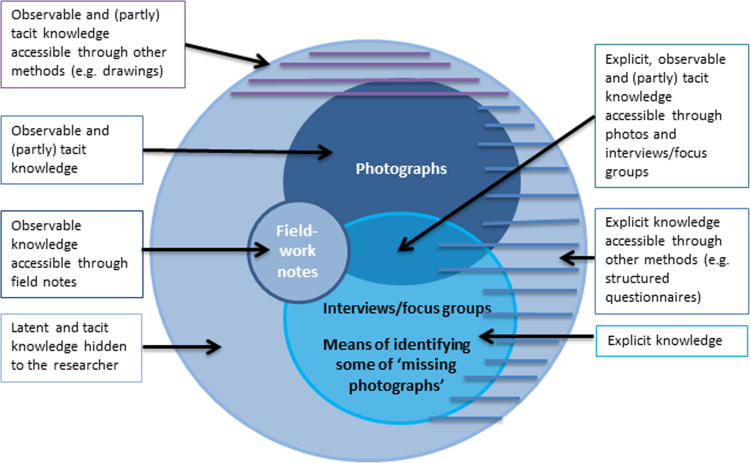
Conceptualisation of the different levels of knowledge that can be accessed through photovoice methods. Adapted from Visser et al. (2005).

This study has shown that the photovoice process enabled participants to become more aware of features of the city contributing to or preventing respect and social inclusion. This highlights one of the strengths of photovoice, wherein the visual element invites participants to actively engage with the photographs, and the discussions offer an opportunity to think critically as a group (Carlson, Engebretson & Chamberlain, 2006). The direct quotes from participants and feedback from attendees resulted in an increased awareness at the individual and the community level as a result of the photovoice process (Evans-Agnew and Rosemberg, 2016, Foster-Fishman et al., 2010, Sanon et al., 2014, Warren, 2005). Further, this study impacted on the individual empowerment, as at the photo-exhibition participants spontaneously engaged with city stakeholders and other attendees in explaining their own photographs and stories (Warren, 2005).Our findings demonstrate how photovoice methods have allowed access to multiple levels of knowledge (Fig. 8). Participants' perceptions are not confined to the photographs that were taken with exploration of the ‘missing photographs’. This emphasises the potential of photographs when complemented by thorough interviews. Sanders (2001) and Visser et al., (2005) explained how different levels of knowledge can be accessed by different research methods. They distinguish between ‘conventional techniques’ (e.g. interviews), and ‘generative techniques’ (e.g. drawings) calling the first ‘explicit knowledge’, providing a context for the second. Thus users can use generative techniques (drawings), and then can discuss their meanings, allowing them to gradually express not only the ‘observable knowledge’ (e.g. the drawing in itself), but some deeper levels of the knowledge. Those levels are the ‘tacit knowledge’ – knowledge difficult to express in words, and ‘latent knowledge’, which individuals are not conscious of.

The combination of different methods in photovoice allowed us to access a broader and deeper level of knowledge, gaining a greater comprehensiveness of understanding of the topic from the participants' point of view (Barbour, 2001). For example, the issue of the ‘missing photos’ would not have emerged without engaging people in the photovoice project. It is therefore a product of bringing the interviews/focus groups and photographs together. Fig. 8 illustrates conceptually the interrelationships between the domains of knowledge that can be accessed through photovoice methods.

A part of the ‘explicit knowledge’ is accessible through other methods (e.g. structured questionnaires) which can be expected to identify some of the issues that emerge from the photographs and interviews. The same applies with a part of the ‘observable and tacit knowledge’ (e.g. accessible through drawings), and from field work notes, which relate to aspects of what participants express or are unwilling to express. However, part of the ‘tacit knowledge’ and the ‘latent knowledge’ are hidden to the researchers and perhaps can never be accessed fully, either through barriers (reluctance, social expectations) or as individuals are not yet aware of it.

### 4.4 Limitations of the study

One potential limitation of the study is the gender imbalance in the sample (males: 7; females=19). A review by Catalani and Minkler (2010) reported that most projects (78%; of 46 studies) recruited a greater number of female groups. This reflects both the greater proportion of women in the population aged 65 and over in the UK (Office for National Statistics, 2015), and that women might be more inclined to participate in community groups. In this study, the guiding principle for recruiting participants was to have a mix of included and less included participants, rather than focusing on gender differences.

Another potential limitation was the limited time available to take photographs (1 week), which had to be balanced against goal of keeping participants engaged and the ideas behind their photographs fresh.

### 4.5 Implications for policy practice and research

This study has suggested that images produced through photovoice can provide highly informative material – when presented through a public exhibition – for engaging a diverse audience including key stakeholders in discussion about aspects of the city that are important to older people. As a result of the photovoice process and photo-exhibition event, this study resulted in an increased awareness at the individual and the community level. As noted, this study aimed to bring older people's views to the attention of city stakeholders so that they could include their concerns in decision making processes for an AFC. At the exhibition event for this study, a number of attendees and participants questioned if the findings could contribute to changing policy and, in response, the mayoral lead for older people stated that the findings would inform the Liverpool joint needs strategic assessment. Whilst it is not possible from this study to identify how this research might impact change in policy, the final phase of the photovoice project (Fig. 1) allowed engagement between participants and city stakeholders in disseminating the findings and in stimulating collective dialogue. Sanon et al. (2014) noted that to promote an impact on policy, alongside the photo-exhibition, is important to follow-up with city stakeholders in various ways. In our study, we have disseminated a report with the findings of older people's enablers and concerns to city stakeholders, and we intend to follow-up to establish the extent to which future AFC strategy includes older people's views. Given the AFC context and the need for older people to be involved in shaping their city, we propose that photovoice is considered as a tool by policy makers and clinical commissioning representatives on a more routine basis, to involve older people in identifying priorities for action, and ensuring that their views are included in decision making and planning processes.

## 5 Conclusions

This paper has reflected upon some of the opportunities, challenges, and solutions in using photovoice methods in our study. Whilst photovoice is practically challenging (e.g. time-consuming), it provides extensive and ‘rich’ data, and an approach well-suited to bringing older people and stakeholders together.

Amongst the opportunities, photovoice approach brought to the surface older people's knowledge in articulating what was most important for their respect and social inclusion in their city. It can therefore enable the articulation of ‘hidden things that are important to people’, which we may not be able ‘to see’ solely through interviews or focus groups. The photographs and accompanying captions presented through a public exhibition, offers a creative way to present, discuss and reflect on the findings in a way that can engage a diverse set of city stakeholders and the wider community. Further, the photo-exhibition aims to advocate policy change around concerns and priority issues identified by participants by bringing those to the attention of city stakeholders, so that participants' views can be included in decision making and planning processes.

The main challenges comprised participants' difficulty in capturing negative and social aspects, and pressure experienced by some participants when taking photographs due to (i) expectations of what is a ‘proper’ photograph, and (ii) the need to obtain consent from subjects. Ways to overcome these can include: training and confidence building with the entire photo-production process, and discussing photographs not taken by participants. Through analysing the application of photovoice for exploring perceptions of respect and social inclusion in cities, our paper has provided important recommendations for researchers on how photovoice methodology can be strengthened in exploring conditions for better health in the urban environment.

## Figures and Tables

**Table:** 

Main questions asked at the interview

● **Tell me why you took this photograph.** ● **Why do you want to share this photograph?** ● **Why is it important for you?** ● **How, in what way does this photograph (name of the building/place in the photograph) make you feel valued and/or part of your community?**
The SHOWeD technique (Wang et al. 1998; Wang & Burris 1997)
● **What do you See here?** ● **What's really Happening here?** ● **How does this relate to Our lives?** ● **Why does this problem, concern, or strength Exist?** ● **What can we Do about it?** ● **What does this photograph represent in terms of positive and negative aspects which support or do not support you to feel valued and part of your community?** ● **How do you think that this photograph/aspect you portrayed may be help to supporting other elder people to age healthily and feel valued and/or part of their community?** ● **Think of two challenges within your neighbourhood/local community/Liverpool that you face every day which constitute a barrier for you to make you feel valued and/or part of the community.** ● **Think of two aspects within your neighbourhood/local community/Liverpool that support you to make you feel valued and/or part of the community.** ● **Think of services/aspects/activities of Liverpool that are inclusive of elder people. Can you give me some examples of this? What type of services would you like to see/do you think that can be inclusive of elder people in the community?** ● **Imagine that there was the possibility to make your neighbourhood/local community/Liverpool a better place for elder people to feel valued and part of their community. In this, imagine that you have all the resources to make it possible. What would you do?** ● **How would like that communicate what is meaningful to you and the results of the projects to people who work in the council and representatives of services for elder people in the city?** Exploring photographs not taken: ● **Are there any photographs that you might have wanted to take but you did not? If yes, can you tell me more about that?** (Hodgetts et al. 2007)
